# COGNITIVE RESERVE, PHYSICAL HEALTH, AND COGNITIVE FUNCTIONING IN OLDER ADULTS

**DOI:** 10.1093/geroni/igac059.2252

**Published:** 2022-12-20

**Authors:** Alyssa MacLean, Jessica Strong

**Affiliations:** University of Prince Edward Island, Charlottetown, Prince Edward Island, Canada; University of Prince Edward Island, Charlottetown, Prince Edward Island, Canada

## Abstract

Prior research has shown positive relationships between cognitive reserve (CR), physical health, and cognition, meaning that higher levels of physical health and CR are associated with higher cognitive functioning and vice versa. A group of community-dwelling older adults (N = 45, mean age = 70.5 years) completed a measure of CR (Life Experiences Questionnaire; LEQ), as well as cognitive tests, with number of physician diagnosed health conditions and number of medications measuring physical health. Initially, we ran correlations with the intention of running a mediation model (physical health factor as the independent variable, LEQ as the mediator, and cognitive test scores as dependent variables). Significant correlations were found between physical health and CR (r = -0.44, p = .01) with a medium effect size, and between CR and some test scores. However, there were no correlations between physical health and cognitive scores. Therefore, using linear regression analyses, the LEQ significantly predicted scores on some tests of executive functioning (DKEFS: Colour-Word Interference Test; Trial 3: F(1,39) = 7.42, p = .010), and processing speed (DKEFS: combined colour naming/reading: F(1,32) = 4.32, p = .046). However, the LEQ did not significantly predict verbal fluency, any set-switching tests, or a set-switching and inhibition test. Additionally, when physical health was added to the model, there was no significant improvement. The results suggest that CR may predict some types of executive functioning test scores, but not other executive functioning tests. Additionally, physical health did not predict cognitive test scores in this sample.

